# A Single-Cell Survey of Cellular Heterogeneity in Human Great Saphenous Veins

**DOI:** 10.3390/cells11172711

**Published:** 2022-08-31

**Authors:** Yiping Sun, Xueqing Hu, Kui Zhang, Man Rao, Pengbin Yin, Ran Dong

**Affiliations:** 1 Department of Cardiac Surgery, Beijing Anzhen Hospital, Capital Medical University, Beijing 100029, China; 2 Beijing Institute of Chinese Medicine, Beijing Hospital of Traditional Chinese Medicine, Capital Medical University, Beijing 100010, China; 3 National Clinical Research Center for Orthopedics Sports Medicine and Rehabilitation, General Hospital of Chinese PLA, Beijing 100039, China

**Keywords:** great saphenous vein, single-cell RNA sequencing, vessel, cell–cell interaction, vein graft failure

## Abstract

Background: The great saphenous vein (GSV) is the most commonly used conduit for coronary arterial bypass graft. However, the status of the GSV, including metabolic dysfunction such as diabetes mellitus (DM) complication, is strongly associated with vein graft failure (VGF). To date, the molecular mechanism underlying VGF remains elusive. Detailed characterization of the cellular components and corresponding expression regulation in GSVs would be of great importance for clinical decision making to reduce VGF. Methods: To this end, we performed single-cell RNA sequencing to delineate cellular heterogeneity in three human GSV samples. Results: Scrutinization of cellular composition and expression revealed cell diversity in human GSVs, particularly endothelial cells (ECs). Our results unraveled that functional adaptation drove great expression differences between venous ECs and valvular ECs. For instance, cell surface receptor *ACKR1* demarcated venous Ecs, whereas *ACRK3/ACKR4* were exclusively expressed by valvular ECs. Differential gene expression analysis suggested that genes highly expressed in venous ECs were mainly involved in vasculature development and regulation of leukocyte adhesion, whereas valvular ECs have more pronounced expression of genes participating in extracellular matrix organization, ossification and platelet degranulation. Of note, pseudo-time trajectory analysis provided in silico evidence indicating that venous ECs, valvular ECs and lymphatic vessels were developmentally connected. Further, valvular ECs might be an importance source for lymphatic vessel differentiation in adults. Additionally, we found a venous EC subset highly expressing *IL6*, which might be associated with undesirable prognosis. Meanwhile, we identified a population of *ANGPTL7^+^* fibroblasts (FBs), which may be profibrotic and involved in insulin resistance in human GSVs. Additionally, our data suggest that immune cells only accounted for a small fraction, most of which were macrophages. By assessing the intertwined remodeling in metabolic dysfunction that potentially increases the gene expression regulatory network in mural cells and leukocytes, we found that transcription factor *KLF9* likely operated a proinflammatory program, inducing the transcription of metallothionein proteins in two mural cell subsets and proinflammatory immune cells. Lastly, cellular communication analysis revealed that proinflammatory signaling, including TRAIL, PVR, CSF and GDF, were uniquely activated in patients with metabolic dysfunction. Conclusions: Our results identified critical cell-specific responses and cellular interactions in GSVs. Beyond serving as a repertoire, this work illustrates multifactorial likelihood of VGF.

## 1. Introduction

Cardiovascular diseases are one of the most prevalent disorders causing global morbidity and mortality, among which occlusive arterial disease severely threatens patients’ health and leads them to a high risk of adverse outcomes [1]. Bypass graft surgery is the most used clinical intervention that efficiently improves the long-term survival of patients suffering occlusive arterial disease, in particular coronary arterial disease (CAD) [2]. By far, the great saphenous vein (GSV) is the major conduit for coronary arterial bypass graft (CABG), with advantages of availability and length [3]. However, GSV graft exposed to an increased pressure flow microenvironment and maladaptation would ultimately develop vein graft failure (VGF) [4]. Risk factors associated with VFG include diabetes, age, chronic kidney disease and metabolic dysfunction such as mellitus (DM) and hypercholesterolemia [5,6,7,8]. The adverse remodeling process in VGF includes endothelium releasing proinflammatory and profibrotic cytokines and growth factors promoting intima hyperplasia formation [9]. Several clinical studies have demonstrated optimized preservation of GSV to maintain normal endothelial function, and integrity of the GSV can reduce VGF [10,11]. Therefore, in-depth characterization of GSV at a cellular level with high resolution is vital to investigate the intervention target to ameliorate VGF.

To this end, single-cell RNA sequencing (scRNA-seq) is a powerful tool to elucidate cellular composition in tissues of interest, the transcriptional profile of an individual cell, cell type-specific expression regulation and cellular communications under different conditions. Recent studies have been dedicated to multiple organs such as brain, heart, liver, lung, kidney and large cardiac vessels in mammalians [12,13,14,15]. Interorgan comparison has revealed that endothelial cells (ECs) are largely influenced by tissue microenvironment and exhibit great differences among ECs of specific origin [14]. Nevertheless, cellular components and their corresponding expression profiles in the GSV or any peripheral vein are not known yet. Herein, by taking advantage of state-of-the-art technology and precious human samples, we aim to establish a comprehensive atlas for the human GSV at single-cell resolution, and hopefully this map would potentially pave a path to the therapeutic avenue for VGF prevention.

## 2. Materials and Methods

### 2.1. Ethical Approval

This study was approved by the Ethics Committee of Beijing Anzhen Hospital. All participants signed their informed consent before being enrolled in our study and the experiments were performed in accordance with the approved study protocol.

### 2.2. Tissue Dissociation and Single-Cell RNA Sequencing

Human great saphenous vein (GSV) was harvested in the procedure of coronary artery bypass graft. Upon excision, GSV was immediately transferred into ice-cold RPMI1640 medium (Thermo Fisher, 11875101, Waltham, MA, USA). GSV was not dilated and was cut into small pieces, rinsed with cold PBS buffer three times and then subjected into collagenase II solution for 30~60 min at 37 °C. Enzymatic reaction was stopped by RPMI1640 medium supplemented with 10% fetal bovine serum (HyClone, SH30071, Logan, UT, USA). Cell debris was removed by centrifugation and supernatant was further filtered by 40 μm strainer. Then, live cells were selected by Miltenyi Biotec Dead cell removal kit (Cat No. 130-090-101, Bergisch Gladbach, Germany). After cell counting, approximately 10,000~15,000 cells were mixed with Chromium^TM^ Single-Cell platform using Chromium^TM^ Single-Cell 3′ Library and Gel Bead Kit v3.1 (10× Genomics, Pleasanton, CA, USA) and single-cell RNA sequencing libraries were constructed according to the manufacturer’s instructions. Finally, all libraries were ultimately sequenced on Illumina Novaseq system.

### 2.3. Bioinformatic Analysis

Raw expression matrices were calculated by CellRanger toolkit (version.5.0 10× Genomics, Pleasanton, CA, USA) via alignment to human genome reference build GRCh38 (Ensembl 88). The low-quality cells were discarded according to following criteria: (1) cells had unique molecular identifier (UMI) less than 800 or more than 20,000; (2) cells had no more than 500 expressed genes; and (3) the mitochondrial genes should account for less than 15% UMI. Subsequently, the batch effect of donors was removed by applying integration workflow wrapped in Seurat (version 4.0, New York, NY, USA). Briefly, we first constructed a reference with finding “anchors” among batches/individuals by reciprocal PCA reduction. Then, we split the combined object by each donor and performed log normalization prior to finding anchors. The UMI count was normalized by the “NormalizeData” function. The top 3000 highly variable genes (HVGs) were calculated with “FindVariableFeatures” and selected for downstream analysis. Data were scaled with the “ScaleData” function, setting the parameter “vars.to.regress” to “percent.mito” and “nUMI”. Principal component analysis (PCA) was performed using the “RunPCA” function with the top 3000 HVGs. To cluster single cells into subsets, we adopted unsupervised graph-based clustering algorithm implemented in Seurat package. “PCElbowPlot” function was used to choose the number of PCs and a shared nearest-neighbor (SNN) graph was constructed using the “FindNeighbors” function with the top 35 PCs. Lastly, cells were clustered by the “FindClusters” and “RunUMAP” functions. “FindAllMarkers” function was applied to detect signature genes for each cluster with setting the parameter “min.pct” to 0.3 and “logfc.threshold” to 0.4. Subsequently, cell clusters were annotated manually to the major cell types according to known markers. Any cluster with multiple markers of two types of cells was manually discarded as a doublet.

### 2.4. Functional Enrichment Analysis

For gene ontology (GO) enrichment analysis, we obtained differential expressed genes (DEGs) for each cell subset by setting log2foldchange as 0.4 and observed in at least 30% of cells via FindAllMarkers function wrapped in Seurat package. Then, enrichment analysis was performed with DEGs using clusterProfiler packages.

The proinflammatory score of endothelial cell subsets was carried out by calculating mean expression of genes involved in cell adhesion that promoted leukocyte infiltration: *SELE, SELP, CSF3, CCL14, IL6, ICAM1* and *HMOX1*.

### 2.5. Transcriptional Noise Analysis

Transcriptional noise analysis was first introduced by Enge et al. [16]. Briefly, for each cell subset, raw UMI counts for each cell were extracted. The cell subsets were downsampled so that all cell subsets had equal number of total UMI. Then, all genes were divided into ten bins based on average expression. Bins at extremity were discarded, and for each rest bin, genes were sorted by coefficient of variation and 10% of genes at the bottom were selected for downstream analysis. Next, down-sampled cell subsets were further reduced to bottom 10% of genes with the lowest coefficient of variation, and UMI counts were square-root-transformed. Lastly, the Euclidean distance was calculated between each cell within a cell subset as a measurement for transcriptional noise.

### 2.6. Pseudotime Trajectory Analysis

The monocle R package (version 2.12.0, Seattle, WA, USA) was used to construct cell-subset pseudo-time trajectory analysis. The cell clusters of interest were selected using the “subset” command of Seurat, and then a CellDataSet object was created with the “as. CellDataSet” function of monocle. After calculating size factors and estimating dispersions, differentially expressed genes among clusters along the trajectory were identified via the “differentialGeneTest” function. To determine significance, we set q-value cutoff as 1E-40 for EC, 1E-25 for FB and mural cells for selecting most differentially expressed genes. “DDRTree” method was adopted in dimension reduction. After cell ordering, the “plot_cell_trajectory” functions were used for visualization.

### 2.7. Regulatory Analysis of Transcription Factors

To infer transcription factor (TF)–target interactions, single-cell regulatory network inference and clustering (SCENIC) algorithm was run with raw expression matrices to identify regulons specifically involving different cell subsets. TF activities (AUCell) for each cell were calculated with motif collections version mc9nr. The significantly upregulated regulon was defined as log fold change of more than 0.1 and adjusted *p*-value < 10^−5^.

In this study, we retrieved targeted genes that were positively correlated with selected TF) from SCENIC output “regulon” file for network analysis visualization. The transcriptional network of TF and predicted target genes were visualized by Complexheatmap package and Cytoscape (v3.8, Seattle, WA, USA).

Additionally, we performed coexpression analysis to further support abovementioned gene expression network analysis. In brief, the log-transformed normalized expression matrix was extracted from cell subset of interest. Then, Pearson correlation was calculated for all genes, and only genes with correlation greater than 0.15 and *p* value lower than 0.05 were kept.

### 2.8. Cell–Cell Interaction Analysis

Cellular crosstalk was calculated with CellChat package [17]. Briefly, we separated object by diabetic mellitus and examined corresponding cell–cell interaction weight, strength and signaling. Only cellular communications in at least 10 cells were kept for downstream analysis. We calculated both outgoing signaling and incoming signaling for both groups. We mainly focused on signaling pathways that were uniquely in GSVs complicated with metabolic dysfunction. For each selected pathway, we analyzed the roles of cell subsets by “netAnalysis_contribution” function. Then, we used “plotGeneExpression” function to visualize ligands and receptors in the selected signaling.

### 2.9. Bulk RNAseq Data Validation

To validate *KLF9* upregulation in diabetes mellitus, we downloaded bulk tissue RNA-sequencing data from GEO database under the accession GSE179231 and GSE162391. GSE179231 was derived from lacrimal gland of three wild-type mice and three diabetic mice. Additionally, GSE162391 was generated from swine coronary artery segments for which 4 nondiabetic and 4 diabetic samples were included.

### 2.10. Data Visualization

Microsoft R Open (version 3.6.1, https://mran.microsoft.com/) (accessed on 5 July 2019) was used. The R packages ggplot2 (version 3.1.0 by Hadley Wickham, Houston, TX, USA), pheatmap (version 1.0.12 by Raivo Klode, Tartu, Estonia) and clusterProfiler (version 3.10.1 by GuangChuang Yu, Guangzhou, China) were used to generate graphs of the data.

### 2.11. Data Availability Statement

The original contributions presented in the study are included in the article/Supplementary Material, further inquiries can be directed to the corresponding authors. The raw data associated with this study has been deposited in the Sequence Read Archive (SRA) under the accession PRJNA835590.

## 3. Results

### 3.1. Transcriptomic Identification of Human Great Saphenous Vein Landscape

**Figure 1:**
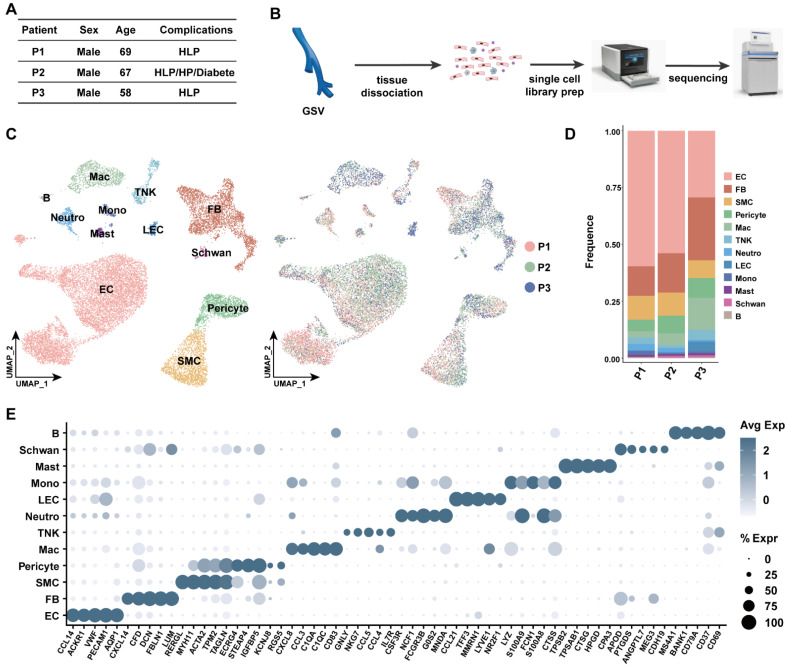
The workflow and overall clustering results. (**A**) Baseline information of patients included in this study. (**B**) The workflow of our study. (**C**) The clustering of all cells identified 12 main cell types in human great saphenous veins. EC: endothelial cells, FB: fibroblasts, SMC: smooth muscle cells, Neutro: neutrophils, Mast: mast cells, Mac: macrophages, Mono: monocytes, LEC: lymphatic endothelial cells, TNK: T cells and natural killer cells, B: B cells. (**D**) Cell composition for each patient was plotted. (**E**) Dotplot shows the signature genes for each cell types. Circle sizes stand for the percentage of cells that expressed genes of interest.

To gain insight on the comprehensive cellular heterogeneity on the human great saphenous vein (GSV), we performed single-cell RNA sequencing (scRNA-seq) on GSVs derived from coronary artery bypass graft (CABG) procedures (Figure 1A). In total, three patients who underwent CABG were included in this study. All patients had hyperlipemia (HLP), and patient P2 was further complicated with metabolic dysfunction, including hypertension (HP) and diabetes mellitus (DM). Patient P2 unfortunately did not survive the perioperative period, while P1 and P3 recovered after a six-month follow-up (Figure 1A). Harvested GSVs were subjected to enzymatic dissociation, and single-cell suspension was subjected to droplet-based 10X Genomics Chromium controller for scRNA-seq library preparation and sequencing (Figure 1B). By applying stringent data filtering (details seen in Methods section), a comprehensive cellular map consisting of 12 cell types was established with 18,957 cells (Figure 1C). No cell type was specific to an individual, reflecting no significant bias in data integration (Figure 1C). The endothelial cells (EC) accounted for the majority of cells in the GSVs, followed by fibroblast (FB), smooth muscle cells (SMC) and pericytes. The most prevalent immune cell type was macrophages (Mac). Lymphoid cells including T cells, natural killer cells and B cells were observed in all samples (Figure 1D). Myeloid lineages such as monocytes (Mono), mast cells (Mast) and neutrophils (Neutro) and a small population of Schwann cells were also detected in all samples (Figure 1D). We selected the five most differentially expressed genes in each cell subset as signatures to further validate the robust clustering (Figure 1E and Table S1).

### 3.2. Venous ECs, Valvular ECs and Lymphatic EsC Had Distinct Expression Programs

**Figure 2:**
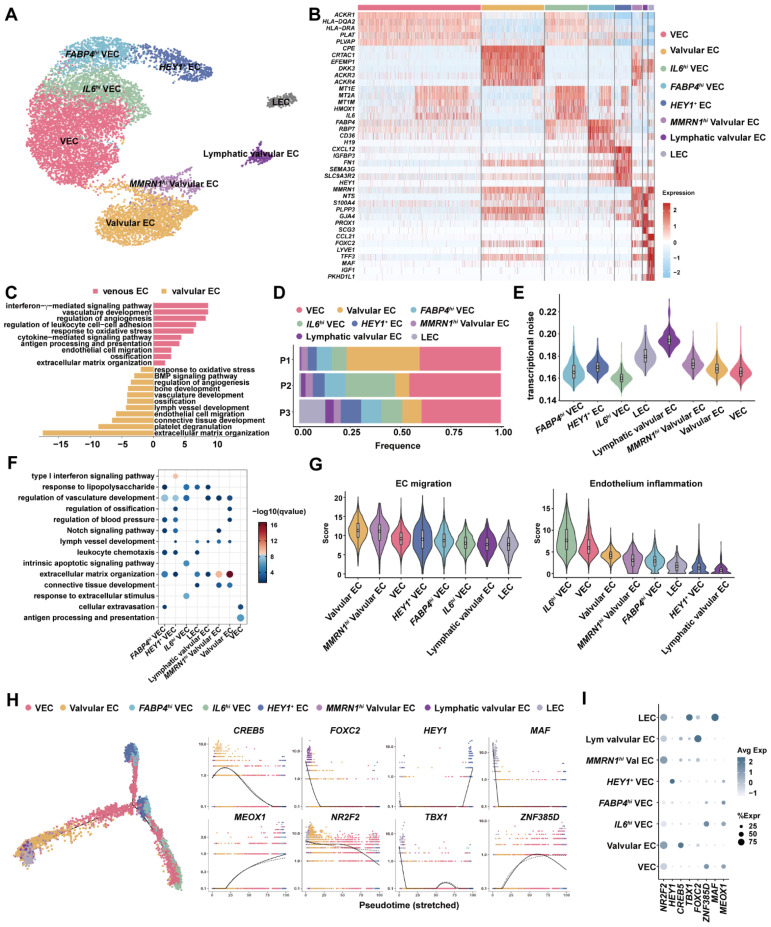
Endothelial cell diversity in human GSVs. (**A**) The UMAP plot of EC cell subsets. (**B**) Heatmap showing the signature genes for each EC subset. (**C**) Gene ontology enrichment of differential expressed gene between venous ECs and valvular ECs. Only upregulated genes were selected for analysis. (**D**) EC cell subset ratio in each patient. This result suggested that *IL6^hi^* VECs were enriched in patients complicated with hypertension and type 2 diabetes. (**E**) Transcriptional noise analysis showing that *IL6^hi^* VECs had the least heterogenous expression. (**F**) Functional enrichment of highly expressed genes for each EC subset. Results infer that valvular EC subsets possessed profound signatures in extracellular matrix organization, whereas venous EC subsets were involved in cell infiltration. (**G**) Comparison of EC migration ability and endothelium proinflammatory score among EC cell subsets. (**H**,**I**) Inference of EC cell subset developmental connection by pseudo-time trajectory analysis. Valvular EC subsets and EC subsets exhibit distinct cell fates. The mast regulators for venous fate (*NR2F2*), arterial fate (*HEY1*), panvalvular fate (*CREB5*), vascular bed differentiation (*MEOX1*), venous EC fate (*ZNF385D*), lymphatic fate (*TBX1*) and lymphatic valvular fate (*FOXC2*) are plotted along trajectories and in cell subsets.

Endothelial cells form the inner lining of all vessels; recently, studies have demonstrated the interorgan and intraorgan cellular plasticity of EC subsets and tissue type contribute more weight in EC heterogeneity [14,18]. The length of the GSVs recruited in our study varied from 3 cm~5 cm; therefore, valves could be digested and pooled in the single-cell suspension (Figure S1A). Some animal-model-based studies have illustrated that lymphatic endothelial cells could be derived from venous ECs; we therefore included lymphatic endothelial cells (LECs) for further analysis [19,20]. Reclustering of ECs and LECs identified eight distinct subsets, including venous ECs (VECs), *IL6^hi^* VECs, *FABP4^hi^* VECs, *HEY1^+^* ECs, LECs and three subsets of valvular ECs (Figure 2A). Valvular identity was determined by *EFNB2* and *ITGA9*, which were necessary for the maintenance of venous valves [21] (Figure S1B). *PROX1* was a canonical marker for lymphatic lineage; *FOXC2* controlled development of lymphatic valve and venous valve in lower limb [22,23] (Figure S1B). Therefore, based on those markers we defined LECs, valvular ECs, *MMRN1^+^* valvular ECs and lymphatic valvular ECs. By calculating the signature gene expression, we found that *ACKR1* was uniquely expressed in all venous ECs while *ACKR3* and *ACKR4* were exclusively expressed in valvular EC subsets (Figure 2B). This was also in consistence with their molecular roles, as *ACKR1* participated in aiding leukocyte infiltration into tissue across vessels, whereas *ACKR3* was involved in thrombosis [24,25]. Moreover, *TEK*, also known as *TIE2*, was highly expressed in *MMRN1^hi^* valvular ECs (Figure S1B). As *TEK* was involved in response to flow shear, we speculated that *MMRN1^hi^* valvular ECs were on the edge of the valvular leaflet [26]. Most approaches in valvular endothelial cells were focused on the aortic valve; little is known on the peripheral vessel valve. Hence, we compared transcriptional profiles of venous ECs (VECs and *IL6^hi^* VECs) and valvular ECs (all three valvular EC subsets). Genes involved in vasculature development for tight and gap junctions (*CLDN5, LRG1, TJP1, ADAMTS9* and *APLNR)*, antigen representation (MHC class II molecules) and cellular response to interferon-γ and stress (*MT1E, MT2A, SOCS3, HMOX1, HIF1A* and *KLF4*) were upregulated in venous ECs, while genes were enriched in extracellular matrix (*FN1, FGF2, DCN, ELN* and *POSTN*), platelet degranulation (*SRGN, APP, MMRN1*, *CD9* and *CLU*), ossification (*BMP4, BMP6*, *MMP2* and *MGP*) and endothelial cell migration (*TEK, ITGB1, VEGFA* and *EFNB2*) were highly expressed in valvular ECs. Therefore, those results reflected that venous endothelial cells were more involved in vasculature development and responses to stimuli or inflammation; however, valvular endothelial cells adapted an expression program in response to shear stress. Notably, genes participating in lymph vessel development such as *PTPN14, FOXC1* and *FOXC2* were highly expressed in valvular ECs (Figure 2C and Table S2).

Next, we assessed cellular composition in each individual, as shown in Figure 2D; nonetheless, the cellular composition was frequently biased in scRNA-seq experiments resulting from many factors including tissue, physiological state of specimen and cell types [27]. To gain an in-depth understanding of EC heterogeneity, we estimated the transcriptional noise of each cell subset, as introduced by Enge et al. [16]. Higher transcriptional noise positively correlated with higher diversity in gene expression regulation. Lymphatic valvular ECs had the most diverse transcriptional noise, while *IL6^hi^* VECs had the most concordant expression variety (Figure 2E). Herein, we inferred that less noise in *IL6^hi^* VECs may reflect its concentrated roles. To associate EC subset function with transcriptomic patterns, we carried out functional enrichment of each EC subset marker list. Results showed that *IL6^hi^* VECs had pronounced apoptotic and inflammatory features (Figure 2F and  Figure S1B). We further compared EC migration and proinflammatory score among EC subsets and pinpointed that *IL6^hi^* VECs had lower expression in EC migration but higher expression in proinflammatory signature (Figure 2G and  Figure S1C). In summary, *IL6^hi^* VECs may be prone to association with adverse remodeling [28].

Pseudo-time analysis is a useful tool to decipher cell fate connection. Herein, we included lymphatic ECs (LECs) into our pseudo-time trajectory, revealing the intertwined relation among those EC subsets: valvular ECs shared tight connections and were not independent from venous ECs in the left branch (Figure 2H and Figure S1D). Additionally, LECs and Lymphatic valvular ECs completely overlapped with valvular ECs in the left branch extremity, indicating that valvular ECs might be a source for lymphangiogenesis in adults (Figure 2H). In a good agreement with previous study, *NR2F2* was a master regulator of venous fate, whilst *HEY1* participated in arterial fate and *TBX1* controlled lymphatic lineage (Figure 2I) [29]. Our results also inferred that *CREB5* was a pan-valvular regulator but had higher expression in vascular valves, while *FOXC2* was mainly involved in lymphatic valve development (Figure 2H,I). Moreover, by taking advantage of single-cell analysis, we demonstrated that transcription factor *ZNF385D* uniquely regulated venous EC differentiation and *MEOX1* influenced vascular bed (neither valve nor lymph vessel) differentiation (Figure 2H,I). In addition, our analysis gave evidence that *MAF* may only impact the lymphatic vascular bed differentiation, which is consistent with the repressive role of *MAF* in blood vascular endothelial cell development [30] (Figure 2H,I).

### 3.3. ANGPTL7^+^ FBs with Antiangiogenic Potential Was Identified in GSV

**Figure 3:**
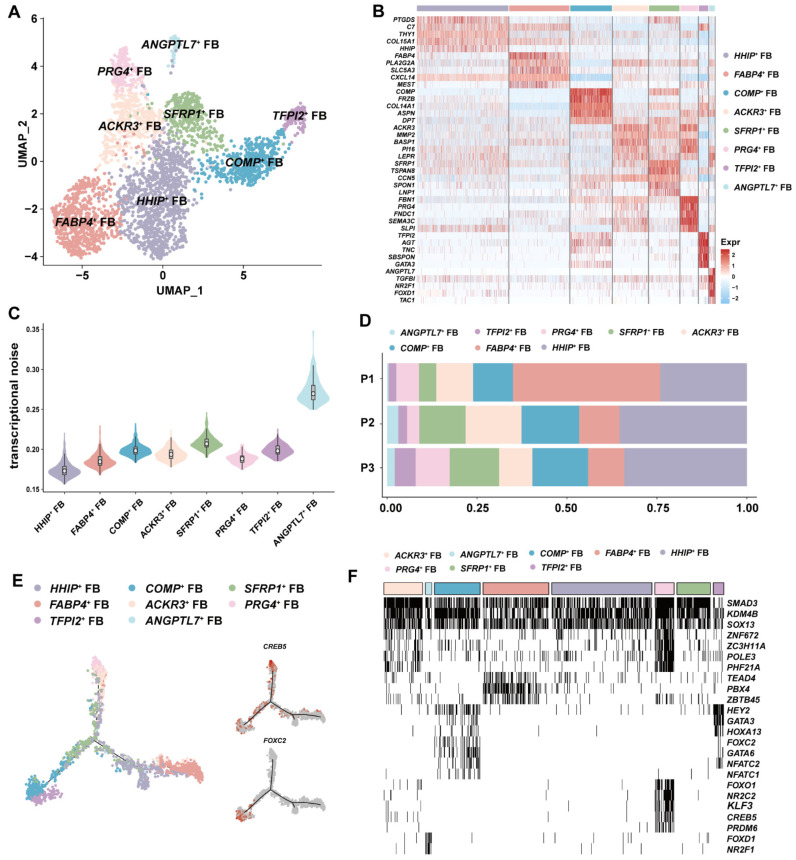
Fibroblast heterogeneity in human GSVs. (**A**) The UMAP plot showing all FB cell subsets. (**B**) Heatmap showing the signature genes for each FB subset. (**C**) Transcriptional noise analysis showing that *ANGPTL7*^+^ FBs had the most variable expression patterns, reflecting a highly heterogenous nature in gene expression. (**D**) Cell ratio comparison identifying that *ANGPTL7*^+^ FBs were enriched in patient P2. (**E**) Pseudo-time analysis revealing that the connection of FB cell subsets was line with their spatial distribution. *CREB5* regulated valvular FB development and *FOXC2* impacted on lymphatic FB. (**F**) Identification of cell-subset-specific regulon by SCENIC. The regulons of *SMAD3*, *KDM4B* and *SOX13* were universally switched on in all FB subsets.

Fibroblasts from vascular beds are typically located in the adventitia and produce extracellular matrix to protect the integrity of veins. Different from arteries, veins contain valves that are populated with fibroblasts [31]. Further clustering analysis divided fibroblasts into eight subsets (Figure 3A). We calculated the signature expression genes and identified a new FB subset, termed *ANGPTL7^+^* FB, which had a very distinct expression compared to other FB subsets (Figure 3B). All FB subsets but *ANGPTL7^+^* FBs have been identified in multiple tissues in mouse and human [32]. To further explore heterogeneity of FB subsets, we assessed the transcriptional noise and showed that *ANGPTL7^+^* FBs had the highest variable expression and were present in all patients (Figure 3C,D). Subsequently, we examined highly expressed genes in *ANGPTL7^+^* FB, which were mainly enriched in the ossification (*TAC1, FZD1* and *ASPN*), negative regulation of phosphorylation (*SOCS3, JUN* and *DDIT4*) and fat cell differentiation (*KLF5, RARRES2* and *ADIRF*) (Figure S2A). *ANGPTL7* can promote lymphatic drainage, and upregulation in hair follicular stem cells suppresses stemness [33]. In addition, *ANGPTL7* exhibited a strong antiangiogenic effect in vitro [34]. A recent mechanistic study reported that overexpression of *ANGPTL7* could upregulate *SOCS3*, which inhibited the phosphorylation of AKT, promoted ERK1/2 phosphorylation and ultimately led to insulin resistance and type 2 diabetic mellitus (T2DM) in mouse [35]. This study also confirmed that ANGPTL7 increased in T2DM patients’ serum. Moreover, in eyes, ANGPTL7 plays an essential role in cross-linked actin networks and overexpression of ANGPTL7 reduces tissue permeability [36,37]. Together with higher fraction of *ANGPTL7^+^* FBs in P2, we speculated that this subset might be associated with dysfunctional endothelium.

In silico trajectory analysis yielded a trifurcated differentiation route for all FB subsets (Figure 3E). As a valvular marker, we speculated that *ACKR3^+^* FBs would be distributed in valves. In addition, previous studies stated that fibroblasts in valves had a differentiation potential reminiscent of mesenchymal stem cells in in vitro culture [38]. The highly expressed *SOX4* and *LEPR* supported that *ACKR3^+^* FBs were probably located in valves (Figure S2B). Overlapped distribution of *ACKR3^+^* FBs and *PRG4^+^* FBs in the upper branch might suggest that both FB subsets were valve-derived and regulated by *CREB5* (Figure 3E). Interestingly, aforementioned results in ECs also indicated that *CREB5* was involved in valve differentiation. We speculated that *COMP^+^* FBs and *TFPI2^+^* FBs at the left branch appeared to belong to lymphatic lineage due to *FOXC2* regulation (Figure 3E). Hence, we speculated that the right branch where *ANGPTL7*^+^ FBs were located might involve cells constituting a vascular bed. To further test our hypothesis, we applied single-cell regulatory network inference and clustering (SCENIC) to investigate cell-type-specific transcriptional control [39]. Our data illustrated that *SMAD3*, *KDM4B* and *SOX13* may act as a universal TF for FB lineage, whereas lymphatic TF *FOXC2* regulated *COMP^+^* FBs and *TFPI2*^+^ FBs and *FOXD1* controlled *ANGPTL7*^+^ FBs (Figure 3F and Figure S2B).

### 3.4. KLF9 Regulated Proinflammatory Programs in Mural Cells

**Figure 4:**
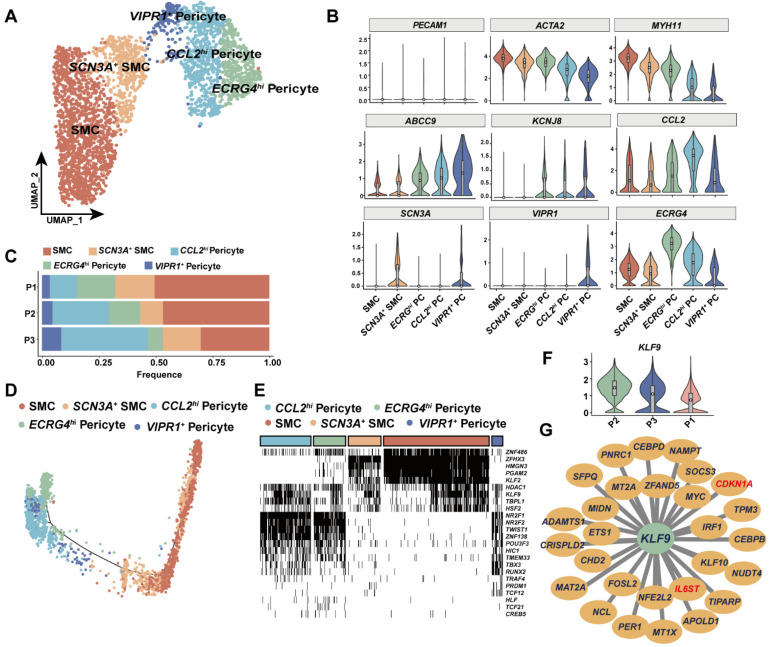
Single-cell analysis of smooth muscle cells and pericytes. (**A**) The UMAP plot of smooth muscle cell (SMC) and pericyte subsets. (**B**) Violin plot of SMC and pericyte conventional markers (upper row) and unique markers identified in our data (bottom row). (**C**) Mural cell composition in each patient is shown. (**D**) Pseudo-time analysis reveals that SMC and pericytes were minimally connected, suggesting that SMC and pericytes might be of different origins. (**E**) Gene expression regulation analysis by SCENIC infers SMC- and pericyte-specific transcription regulator. Transcription factors (TFs) including *PGAM2* and *KLF2* controlled express programs in SMC while TFs such as *NR2F1* and *ZNF138* impacted on pericyte transcription. *KLF9* regulon was specifically active in patient complicated with hypertension and diabetes. (**F**) Violin plot of *KLF9* expression in descending order, showing that *KLF9* was upregulated in P2. The patient was complicated with hypertension and diabetes. (**G**) Network analysis shows direct targets of *KLF9* in both SMCs and pericytes.

Smooth muscle cells (SMC) and pericytes are mural cells that play different roles in vasculature. SMCs keep contractile function for fluid flow, whilst pericytes stabilize the vessel wall [40]. We further clustered SMCs and pericytes into five subsets, including *SCN3A*^+^ SMC and *VIRP1*^+^ pericytes (Figure 4A). In the absence of *PECAM1* (also known as *CD31*), canonical SMC marker *ACTA2* and *MYH11* were highly expressed in SMCs and conventional pericyte markers (*ABCC9* and *KCNJ8*) identified the pericyte population (Figure 4B). *SCN3A* encodes a sodium channel protein that is highly expressed in the brain and affects neuron migration [41]. *VIPR1* produces a small vasoactive neuropeptide. *SCN3A*^+^ SMC and *VIRP1*^+^ pericytes accounted for a small fraction of mural cells (Figure 4C and Figure S3). Additionally, we identified a group of pericytes that highly expressed *CCL2*, which could facilitate monocyte infiltration via *CCR2* signaling (Figure 4B). To further understand the developmental connections among those cells, we performed pseudo-time trajectory analysis. We found that SMC subsets and pericytes were independent on trajectory, thus suggesting that they may not be derived from a common progenitor (Figure 4D). Subsequently, we applied SCENIC analysis to investigate gene expression regulation in those SMCs and pericytes. Indeed, regulatory analysis supported that SMCs and pericytes were developmentally distinct cell types that were under unique transcriptomic control (Figure 4E). Unexpectedly, we identified that the *KLF9* regulon was active in patient P2, who was complicated with metabolic dysfunction (Figure 4E). Consistently, *KLF9* had a higher expression level in mural cells in patient P2 (Figure 4F). This finding was supported by the observation that an increased *KLF9* expression was also seen in diabetic mice and swine models (GSE179231 and GSE162391, respectively, Figure S4), indicating that *KLF9* upregulation was probably associated with metabolic dysfunction, including DM. We further carried out gene expression analysis by SCENIC to investigate KLF9 regulated genes in mural cells. In addition, we only focused on genes which were positively regulated by KLF9. In parallel, we performed gene coexpression analysis in all mural cells. Combining results from both methods, we constructed a gene regulation network (Figure 4G, Table S3). Our results illustrated that *IL6ST*, a signaling transducer for various inflammatory cytokines and risk gene for CAD, was a direct target downstream of *KLF9* [42] (Figure 4G). Furthermore, another target, *CDKN1A*, in the downstream of *KLF9*, reflected that mural cells were under cell-cycle arrest (Figure 4G). In conclusion, our analysis inferred that upregulation of *KLF9* may increase the likelihood of adverse remodeling via facilitating inflammation and hindering cell proliferation and arterialization after CABG.

### 3.5. Investigation of Immune Cell Diversity in Human GSV

**Figure 5:**
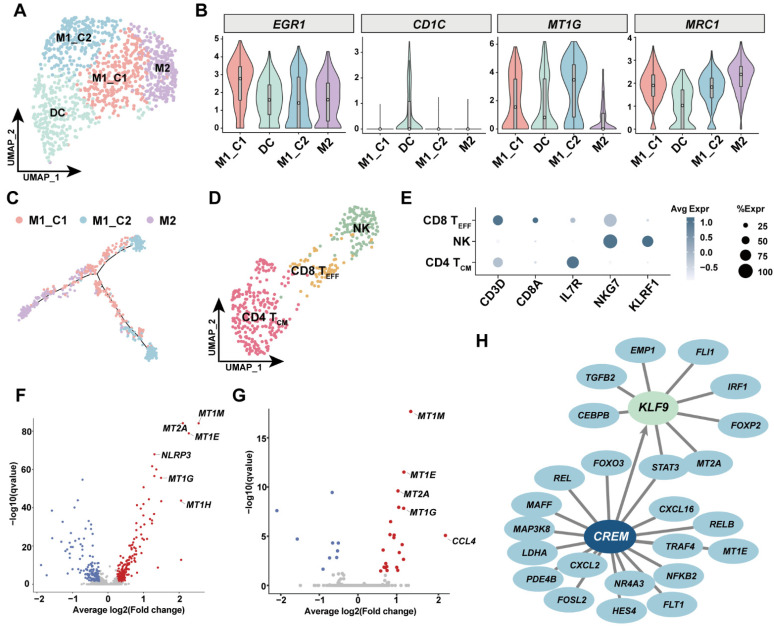
Single-cell analysis of major immune cells in human GSVs. (**A**) The UMAP plot of macrophages and dendritic cells in this study. DC: dendritic cells; M1_C1: proinflammatory M1 macrophage cluster 1; M1_C2: proinflammatory M1 macrophage cluster 2; M2: proreparative M2 macrophages. (**B**) Violin plot of signature genes for macrophages and DC subsets. (**C**) Trajectory analysis of three macrophage subsets. (**D**) The UMAP plot of T-cell subsets and natural killer cells. NK: natural killer cells. CD8 T_EFF_: effector CD8 T cells. CD4 T_CM_: central memory CD4 T cells. (**E**) Dot-plot of signature genes for T-cell and NK subsets. (**F**) Volcano plot showing the differentially expressed genes (DEG) in macrophages and DC between diabetic patients and nondiabetic patients. Red dots represent upregulated genes in diabetic patient (P2). (**G**) Volcano plot showing DEG in lymphocytes between P2 and P1P3. Red dots are genes upregulated in P2. (**H**) Network analysis inferred from SCENIC demonstrates that transcription factors *CREM* and *KLF9* were involved in proinflammatory response in lymphocytes. *KLF9* was one of the downstream targets of *CREM*.

The immune cell compartment is not fully characterized in peripheral vessels; hereby, we performed reclustering of macrophages and T cells. Our results indicated that immune cells accounted for less than 1% cell composition in human GSV. Macrophages could be further divided into three subsets of macrophage and a group of dendritic cells (Figure 5A). Proinflammatory M1 macrophages had a specific subgroup, designated as M1_C2, which highly expressed metallothionein protein *MT1G*. While for M2 macrophages it was highly expressed *MRC1* (Figure 5B). As the origin of dendritic cells is controversial, we only included M2, M1_C1 and M1_C2 or pseudo-time trajectory analysis. The trajectory of the three macrophage subsets showed that the pro-repair M2 macrophage partially overlapped with M1_C1, while M1_C2 was likely a terminal differentiated status derived from M1_C1 macrophages (Figure 5C). Moreover, M1_C2 had a tendency to be enriched in patient P2 (Figure S5). Reclustering of T cells only defined central memory CD4^+^ T cells and effector CD8^+^ T cells (Figure 5D,E). As patient P2 failed to survive after CABG, we then compared gene expression between patient P2 and patients P1/P3 to investigate possible molecular alterations related to this failure. Both macrophages and T cells showed alleviated metallothionein protein expression in GSVs affected with metabolic dysfunction (Figure 5F,G). Moreover, macrophages in GSVs affected with metabolic dysfunction upregulated *NLRP3*, the key regulator of NLRP inflammasome, indicating that metallothionein upregulation was in line with augmented inflammation. To gain insight in such proinflammatory expression programs, we constructed a TF-target network in immune cells and further identified *CREM* and *KLF9* as possible master regulators by SCENIC analysis (Figure 5H).

### 3.6. Cell–Cell Interaction Analysis Unraveled Proinflammatory Programs under Metabolic Complications

**Figure 6:**
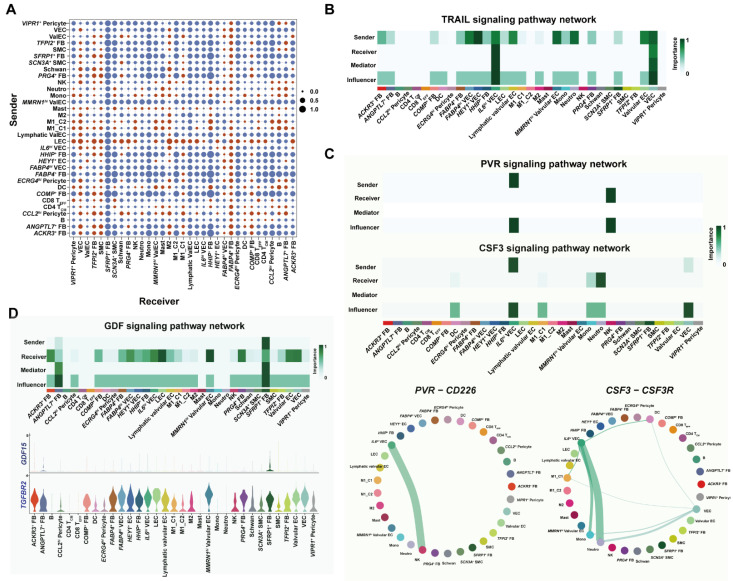
Cell–cell interaction analysis of all cell subsets in human GSV. (**A**) The interaction strengths among all cell subsets were compared between diabetic patient (P2) and nondiabetic patients. Red dots stand for interaction strength that was increased in P2, whereas blue dots are decreased interaction in P2. (**B**) The plot demonstrates the signaling role of cell subsets in TRAIL signaling, and results imply that *IL6^hi^* VECs were major receivers for this inflammatory pathway. (**C**) Heatmap represents roles of PVR and CSF signaling that were specific to diabetic venous EC subsets. Circular plot at the bottom shows the signaling strengths of ligand–receptor in PVR and CSF3 signaling pathway. (**D**) Heatmap represents roles of GDF signaling that was enriched in *ANGPTL7*^+^ FB. Violin plot at the bottom shows the GDF signaling ligand (*GDF15*)–receptor (*TGFBR2*) in all cell subsets.

Clinical studies have demonstrated a strong association between metabolic diseases including DM and VGF; therefore, a rewiring of cell–cell interaction in such conditions would ignite mechanistic studies for translational treatment to prevent VGF [43,44]. We adopted the CellChat toolkit and calculated the interaction weight and strength of all cell subsets in the patient with DM/HP (herein called GSV with DM) and non-DM patients separately. Of note, we found that venous ECs and SMCs complicated with DM received increased signals from most of the other cell subsets (Figure 6A). To further explore such altered communication, we compared signaling pathways between the two groups, revealing that TRAIL, CSF, PVR, OSM and GDF signals were uniquely presented in GSV with DM, most of which were associated with inflammatory responses (Figure S6). TRAIL signaling was increased in many cell types in GSV with DM; we thereafter implemented net analysis. This suggests that most EC and FB subsets were involved as ligand donors, whilst *IL6^hi^* ECs were major receivers (Figure 6B). We then analyzed the signaling pathways enriched in EC subsets. The *PVR-CD226* interacting ligand–receptor pair from PVR signaling mainly involved *IL6^hi^* ECs and cytotoxic NK cells, this interaction may be responsible for endothelial cell dysfunction (Figure 6C). Moreover, *IL6^hi^* ECs also can send *CSF3* and interact with proinflammatory M1 macrophages, monocytes and neutrophils via *CSF3R* (Figure 6C). This interaction could further facilitate adverse remodeling in GSVs. GDF signaling was specifically involved in FB subsets with DM (Figure 6D and Figure S6). *GDF15*, the only ligand detected in our study, was associated with systematic inflammation, DM and obesity [45,46,47,48]. Our results showed that *ANGPTL7^+^* FBs and *SFRP1^+^* FBs expressed *GDF15* and acted on a variety of cells (Figure 6D). *GDF15-TGFBR2* interaction was reported to induce apoptosis in vitro; therefore, we speculated that *ANGPTL7^+^* FBs further induced endothelium damages under DM [48]. Taken together, metabolic dysfunctions, including DM, would increase the likelihood of VGF through a multifaceted interaction involving proinflammatory EC, FB, cytotoxic NK and myeloid cell subsets.

## 4. Discussion

The GSV is the major vessel donor for CABG and other occlusive arterial diseases, although recent studies have demonstrated that artery-derived donors may result in better outcomes [49]. Here, for the first time, we have leveraged state-of-the-art techniques to unravel the heterogeneity of the human great saphenous vein. In contrast to other tissues, ECs in GSVs exhibited highly heterogenous phenotypes. The observation that an artery-like *HEY1*^+^ EC in vein was identified further highlighted that vessels are highly plastic. Considering that valve formation requires arterial gene expression, we reasonably assumed that coexistence of arterial ECs in vein probably gave rise to valves and to better adaptation when arterialization occurred [21]. Further, as stated by recent studies that the tissue microenvironment played essential roles in shaping cell-type-specific expression, we therefore inferred the cell population in valves by checking the valvular marker *ACKR3* [50,51]. Likewise, *SCN3A* was expressed in both some SMC and pericytes; we reasonably assumed that *SCN3A*^+^ SMC and *VIRP1*^+^ pericytes had similar tissue locations. Therefore, our results added valuable evidence to diverse cellular heterogeneity in vessels.

More importantly, our results provided clues at a single-cell resolution about to which extent metabolic dysfunction impacted peripheral veins. Although only one sample was affected with metabolic dysfunction, upregulation of *IL6* and *KLF9* had been supported with other metabolically dysfunctional tissues in humans and other mammalians [52]. In addition, we compared *IL6* expression among three patients, and P2 had the highest *IL6* expression (Figure S7). We therefore concluded that multifactorial adaptation under metabolic dysfunction in GSVs likely orchestrated adverse remodeling as follows: *IL6^hi^* VEC content had the least transcriptional noise and most-highly expressed *CDKN1A* and other proinflammatory molecules, indicating that *IL6^hi^* VECs had decreased potential in proliferation or angiogenesis and thereby probably dampened graft survival. As *FOXD1* could promote pulmonary and kidney fibrosis, we therefore suspected that *ANGPTL7*^+^ FBs were profibrotic FBs across the vessel wall [53,54]. Hence, together with profibrotic *ANGPTL7^+^* FBs, graft appeared to fail dramatically after CABG. Nevertheless, our conclusion should be strengthened via in vitro or in vivo validations with vascular tissues. Immune cells had significantly higher metallothionein proteins, which are induced by many stimuli, including cytokines and oxidative stress, and may function as a negative regulation for apoptosis in cancer [55]. An scRNA-seq study in human adipose tissues reported that metallothionein proteins were positively associated with adipose dysfunction and potentially insulin resistance [56]. Furthermore, *KLF9* was specifically activated in GSVs with metabolic dysfunction, regulating the proinflammatory program in mural cells and proinflammatory immune cells, including M1 macrophage and cytotoxic effector T cells. Combined with a recent mechanism study for *KLF9* in dexamethasone-induced DM, we believe that *KLF9* deserves consideration as a preventative therapeutic target for ameliorating endothelium damage in metabolic dysfunction [57]. Cell–cell interaction analysis deduced unique cell communications in GSVs affected with metabolic dysfunction and particularly highlighted several signaling networks coordinating diabetic milieu. In fact, this result pointed that *IL6^hi^* VECs and *ANGPTL7^+^* FBs could serve as central hubs to transmit inflammatory signals and facilitate leukocyte infiltration and endothelium dysfunction. Despite these novel findings in our study, due to technical difficulties in cell isolation and the unavailability of long human GSV specimens, the major limitation was limited samples, especially for veins affected with metabolic dysfunction. Increased sample size would empower our findings for experimental validations. Hopefully, with ongoing scRNA-seq data, we hope that our current observation could be validated by other datasets and ultimately stimulate a series of mechanism studies and pave a path to novel translational approaches.

## Data Availability

The original contributions presented in the study are included in the article/Supplementary Material, further inquiries can be directed to the corresponding authors. The raw data associated with this study has been deposited in the Sequence Read Archive (SRA) under the accession PRJNA835590.

## Supplementary Materials

The following supporting information can be downloaded at: https://www.mdpi.com/article/10.3390/cells11172711/s1, Figure S1: A: representative picture of human great saphenous vein enrolled in our study. B. Violin plot of endothelial cells canonical genes. C. Endothelium inflammation score comparisons among all defined cell subsets. D. pseudo-time trajectory analysis of EC subsets. The trajectory was split by EC subsets on the left and shown as in pseudotime on the right. Figure S2: A: GO enrichment for upregulated genes expressed in ANGPTL7+ FB. B. Violin plot of mesenchymal cell canonical genes and FOXD1. Figure S3: Split UMAP plots of mural cells. Figure S4: Klf9 expression was upregulated in diabetic mice (A) and diabetic pigs (B). Figure S5: Cell ratio assessment for immune cells. Figure S6: The outgoing signals for each cell subsets in patient affected with diabetes mellitus. and non-diabetic patients were plotted separately. Cells of the same type were shown as the same color. DM: diabetes mellitus. Non_DM: non-diabetic patients. Results showed that CSF3, GDF, OSM, PVR and TRAIL signaling pathways were specifically found in patients with DM. Figure S7: Comparison IL6 expression between DM and non-DM in all cells and endothelial cells. Table S1: Signature expression of each cell subset. Table S2: DEG analysis of venous EC and valvular EC. Pct.1: venous EC, pct2. valvular EC. Table S3: co-expressed genes with KLF9 in mural cells.
