# Supplementary material for: A Single-Cell Survey of Cellular Heterogeneity in Human Great Saphenous Veins
Source: Cells. 2022 Aug 31;11(17):2711. doi: 10.3390/cells11172711 (PMC9454806; doi:10.3390/cells11172711)
Supplement: Supplementary file 1 [file cells-11-02711-s001.zip › Revised Supplementary Figure legends.pdf]

Supplementary Figure Legends

Figure S1: A: representative picture of human great saphenous vein enrolled in our study. B. Violin plot of endothelial cells canonical genes. C. Endothelium inflammation score comparisons among all defined cell subsets. D. pseudo-time trajectory analysis of EC subsets. The trajectory was split by EC subsets on the left and shown as in pseudotime on the right.

Figure S2: A: GO enrichment for upregulated genes expressed in *ANGPTL7*$^{+}$ FB. B. Violin plot of mesenchymal cell canonical genes and *FOXD1*.

Figure S3: Split UMAP plots of mural cells.

Figure S4: *Klf9* expression was upregulated in diabetic mice (A) and diabetic pigs (B).

Figure S5: Cell ratio assessment for immune cells.

Figure S6: The outgoing signals for each cell subsets in patient affected with diabetes mellitus. and non-diabetic patients were plotted separately. Cells of the same type were shown as the same color. DM: diabetes mellitus. Non_DM: non-diabetic patients. Results showed that CSF3, GDF, OSM, PVR and TRAIL signaling pathways were specifically found in patients with DM.

Figure S7: Comparison *IL6* expression between DM and non-DM in all cells and endothelial cells.
