# Supplementary figures and images for: A Single-Cell Survey of Cellular Heterogeneity in Human Great Saphenous Veins
Source: Cells. 2022 Aug 31;11(17):2711. doi: 10.3390/cells11172711 (PMC9454806; doi:10.3390/cells11172711)

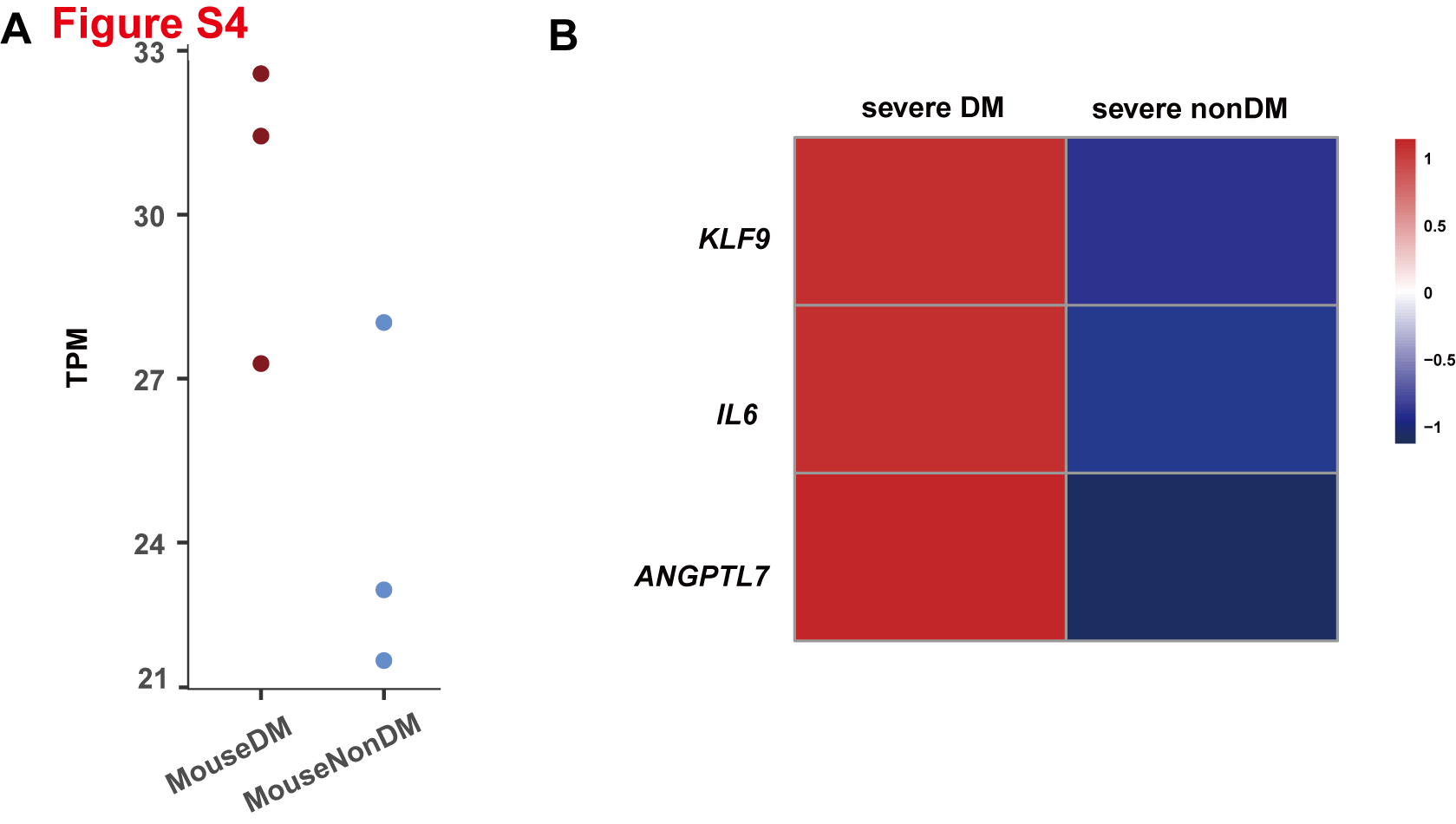

Supplement: Supplementary file 1 [file cells-11-02711-s001.zip › FigS4-KLF9.tif]

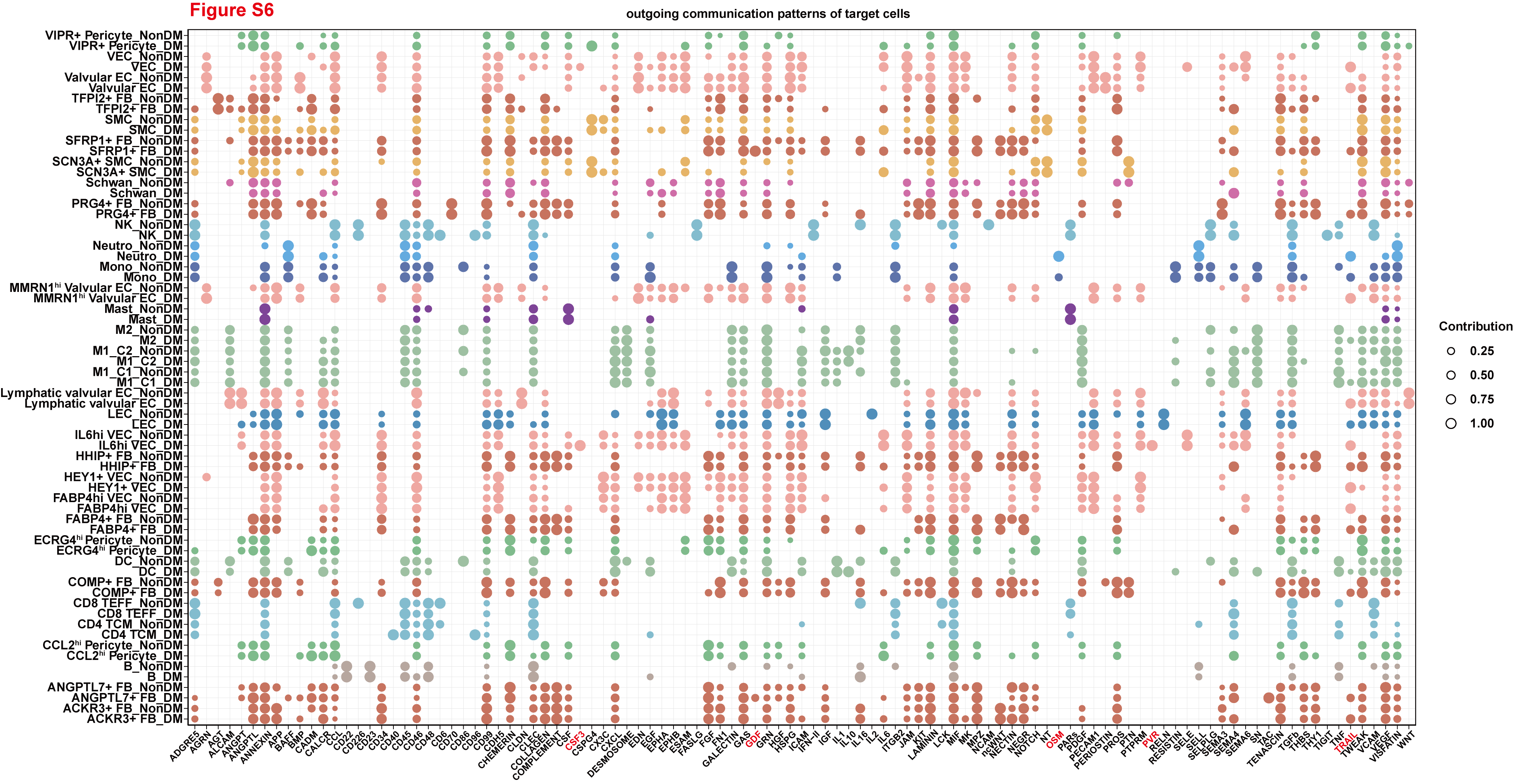

Supplement: Supplementary file 1 [file cells-11-02711-s001.zip › FigS6-interaction.tif]

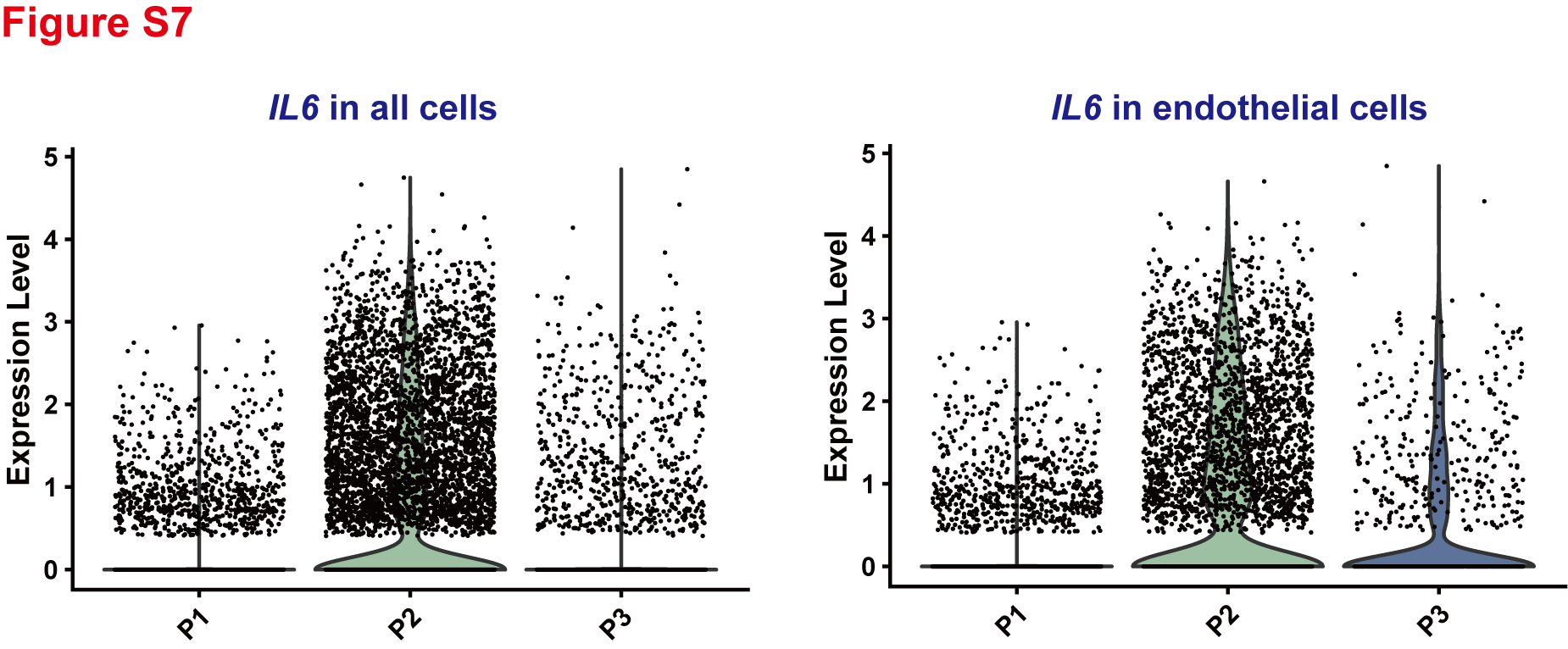

Supplement: Supplementary file 1 [file cells-11-02711-s001.zip › FigS7.tif]

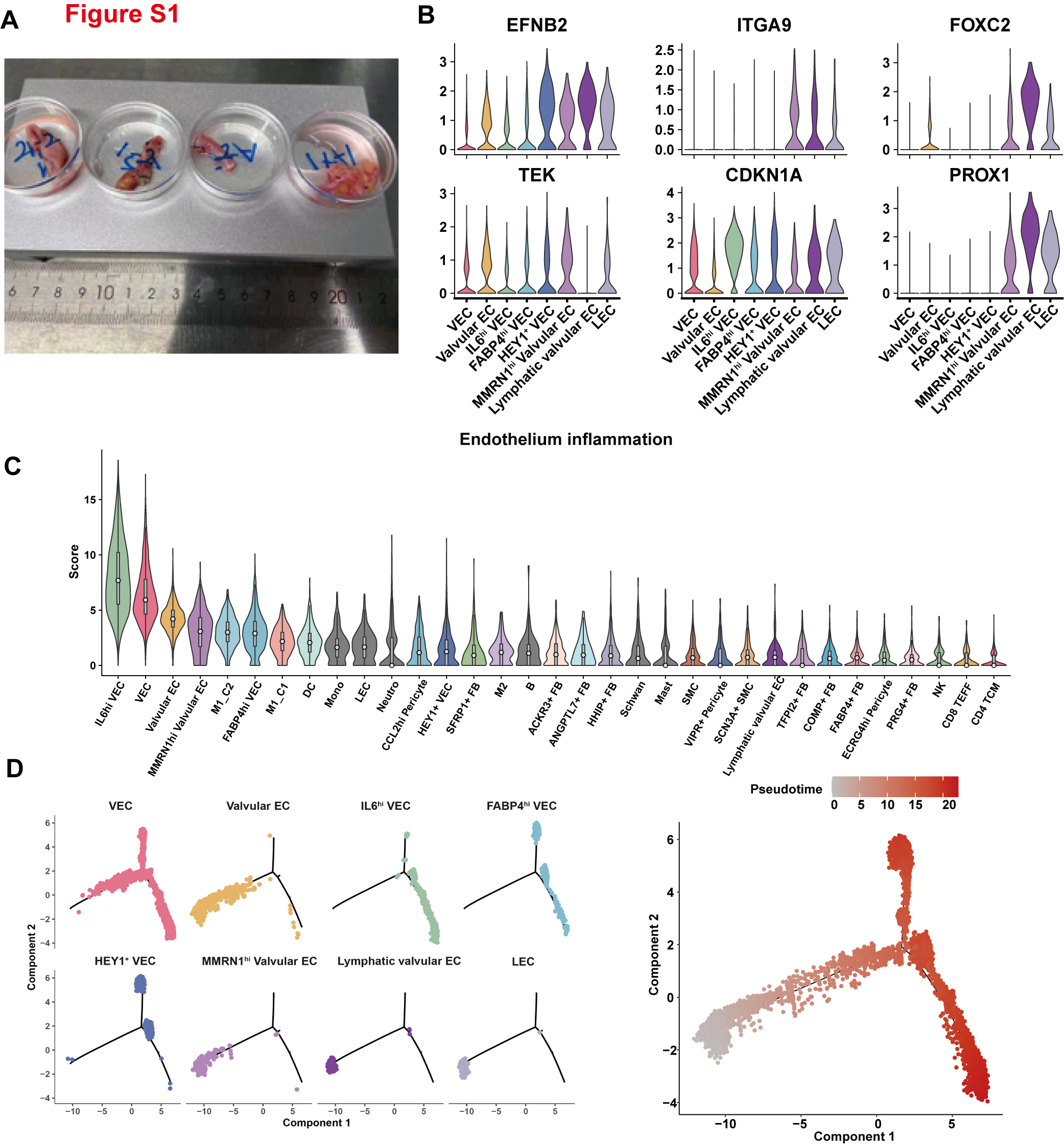

Supplement: Supplementary file 1 [file cells-11-02711-s001.zip › Rev-FigS1-EC.tif]

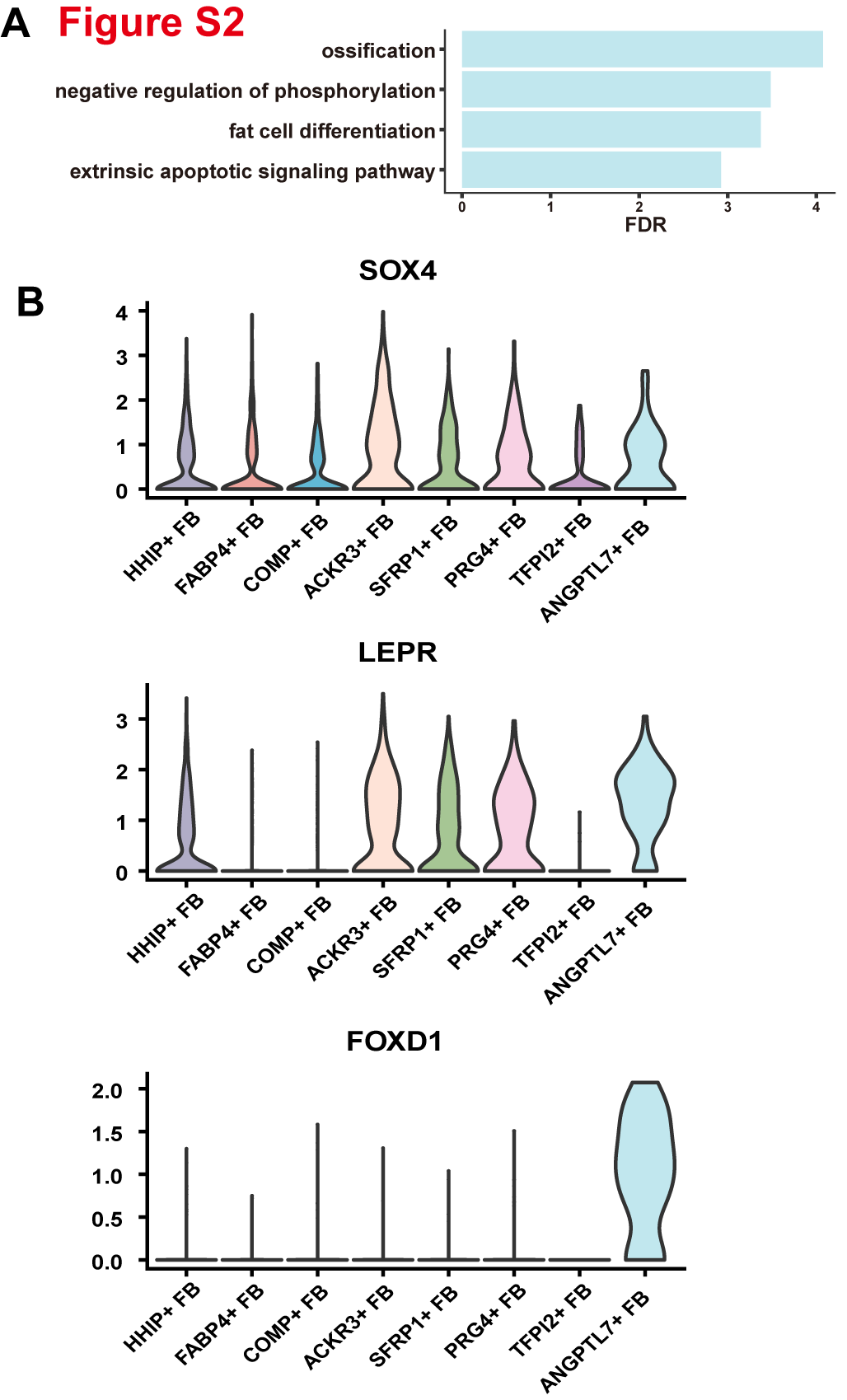

Supplement: Supplementary file 1 [file cells-11-02711-s001.zip › Rev-FigS2-FB.tif]

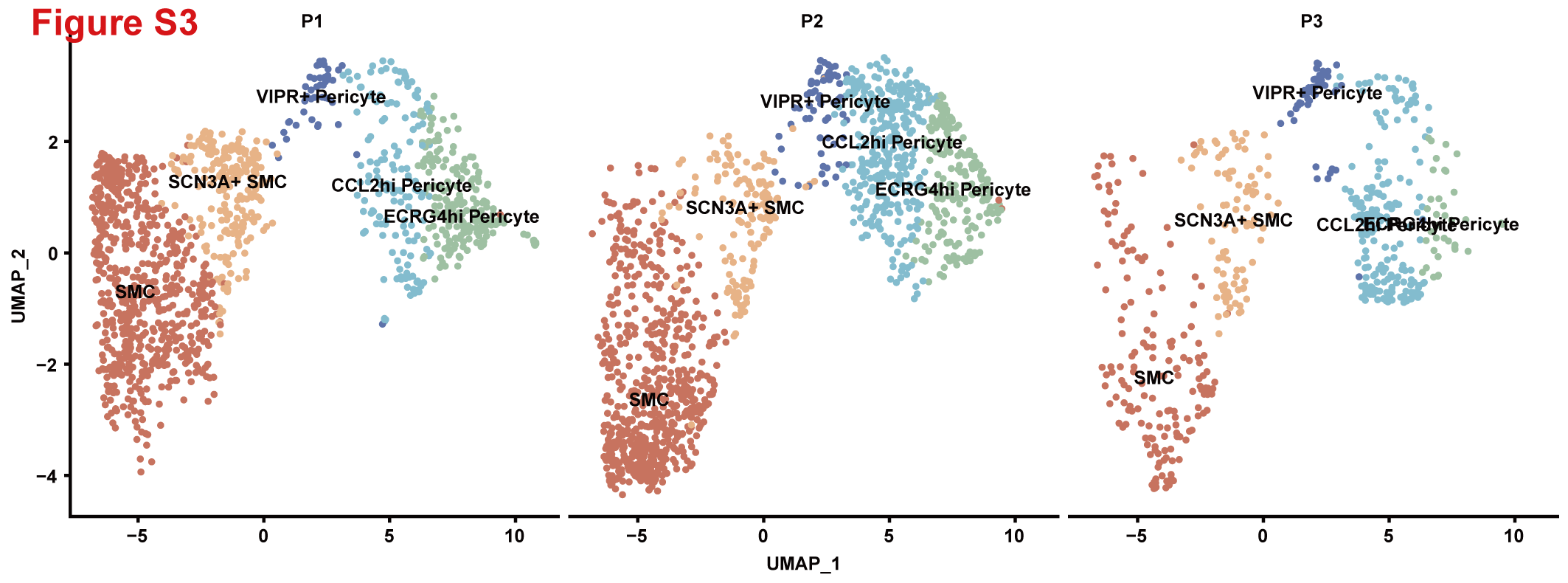

Supplement: Supplementary file 1 [file cells-11-02711-s001.zip › Rev-FigS3-MuralCells.tif]

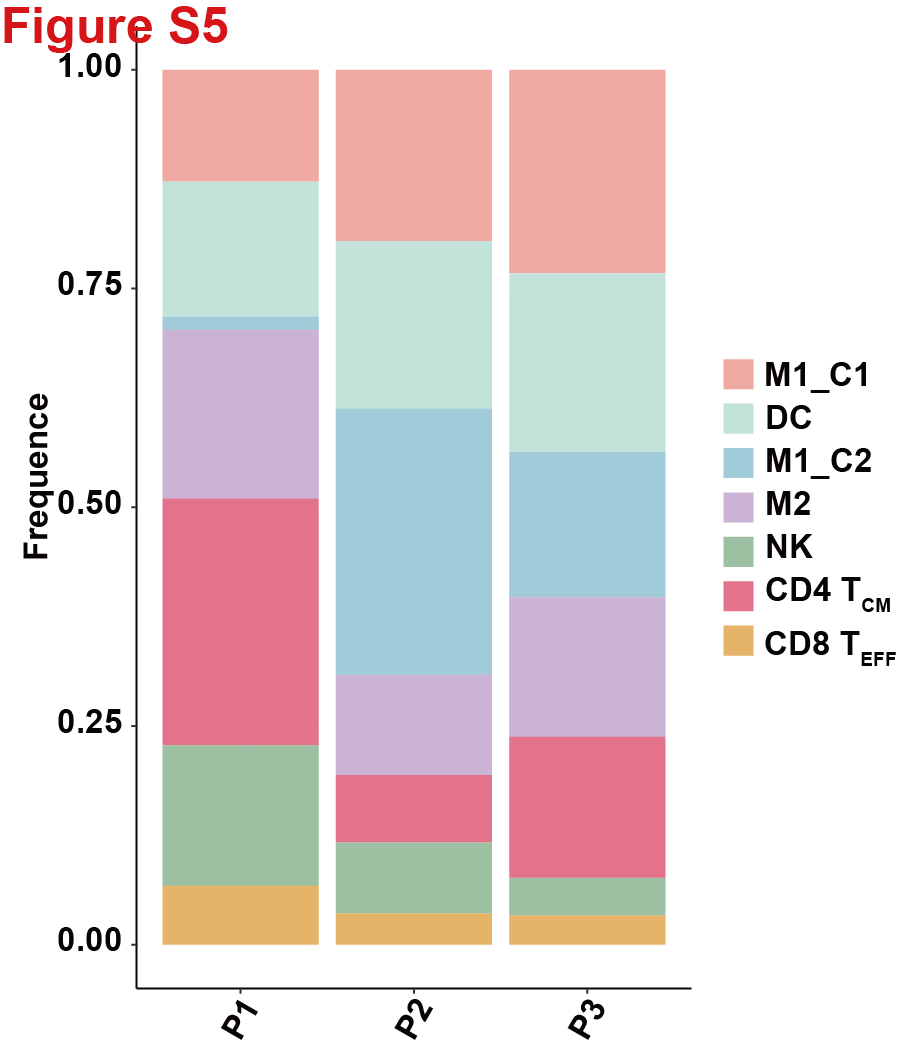

Supplement: Supplementary file 1 [file cells-11-02711-s001.zip › Rev-FigureS5-immuneCells.png]
